# Five-Year Outcomes of Endovascular Aortoiliac Reconstruction in Complex Lesions: A Retrospective Single-Center Study

**DOI:** 10.3390/jcm15145409

**Published:** 2026-07-10

**Authors:** Madeleine Luther, Tim Wittig, Birte Winther, Axel Fischer, Sandra Düsing, Daniela Branzan, Markus Doß, Andrej Schmidt, Dierk Scheinert, Sabine Steiner

**Affiliations:** 1 Department of Angiology, University Hospital Leipzig, 04103 Leipzig, Germany; madeleine.luther@medizin.uni-leipzig.de (M.L.); birte.winther@medizin.uni-leipzig.de (B.W.); axel.fischer@medizin.uni-leipzig.de (A.F.); sandra.duesing@medizin.uni-leipzig.de (S.D.); andrej.schmidt@medizin.uni-leipzig.de (A.S.); dierk.scheinert@medizin.uni-leipzig.de (D.S.); 2 Helmholtz Institute for Metabolic, Obesity and Vascular Research (HI-MAG), Helmholtz Center Munich, University of Leipzig and University Hospital Leipzig, 04103 Leipzig, Germany; sabine.m.steiner@meduniwien.ac.at; 3 Clinic and Polyclinic for Vascular and Endovascular Surgery—Klinikum Rechts der Isar, TUM University Hospital, Technical University of Munich, 81675 Munich, Germany; daniela.branzan@mri.tum.de; 4 Department of Vascular Surgery, University Hospital Leipzig, 04103 Leipzig, Germany; markus.doss@medizin.uni-leipzig.de; 5 Division of Angiology, Department of Medicine II, Medical University Vienna, 1090 Vienna, Austria

**Keywords:** peripheral arterial disease, endovascular therapy, aortoiliac occlusive disease, kissing stenting, Covered Endovascular Reconstruction of the Aortic Bifurcation

## Abstract

**Background**: The objective of this study was to evaluate the long-term safety and efficacy of endovascular therapy (EVT) in complex aortoiliac occlusive disease (AIOD) treated with aortic bifurcation reconstruction using the kissing stent or Covered Endovascular Reconstruction of the Aortic Bifurcation (CERAB) technique. **Methods**: This retrospective single-center study included 201 patients (79.6% ASA stage ≥3) with symptomatic TASC-II C/D AIOD who underwent EVT between 2010 and 2019. Endpoints included technical success and procedural complications. Outcomes assessed over a 5-year follow-up included primary and secondary patency rates, major limb amputation, and survival. Kaplan–Meier (KM) estimates and Cox regression were used for analysis. **Results**: The cohort had a mean age of 61.9 ± 9.7 years, included 61 female patients (30.3%), and showed a low prevalence of obesity (body mass index >30: 7.5%), while cardiovascular risk factors were highly prevalent, including hypertension in 81.1% and hyperlipidemia in 79.6% of patients. Technical success was achieved in 96.0% (193/201), with 87.1% (175/201) of patients treated with kissing stents and 12.9% (26/201) with the CERAB technique. Overall, major complications were reported in 6.5% (13/201) and access site-related complications in 3.0% (6/201) of cases. Five-year KM estimates were: 72.1% for primary patency, 89.6% for secondary patency, and 79.6% for overall survival. No significant difference in primary patency was observed between techniques (*p* = 0.55), and one case of major limb amputation was reported after 5 years. **Conclusions**: EVT using the kissing stent or CERAB technique is a durable and safe treatment for complex AIOD in high-risk patients over 5 years, with favorable outcomes and low periinterventional morbidity and mortality.

## 1. Introduction

Aortoiliac occlusive disease (AIOD) is present in approximately half of patients with peripheral artery disease (PAD) [1]. In its advanced form, AIOD can lead to complete occlusion of the aortoiliac segment, manifesting as bilateral lower limb claudication, lower limb muscle atrophy, and impaired sexual function, also known as Leriche syndrome [2]. Traditionally, aortobifemoral bypass surgery has been the standard of care for complex lesions classified as TransAtlantic Inter-Society Consensus (TASC) II C and D, due to favorable long-term patency [3]. In recent years, however, endovascular therapy (EVT) has emerged as a less invasive alternative, offering lower short-term morbidity and mortality, particularly beneficial in high-risk patients unsuitable for open surgery [4]. As the PAD patient population becomes more complex and comorbidities increase, renewed emphasis is placed on EVT even in high-grade AIOD, classified as TASC-II C/D [5].

Covered stents have become the preferred option for complex aortoiliac interventions due to their favorable patency rates [6,7]. Among EVT techniques, the kissing stent method, which involves the simultaneous deployment of two covered stents at the aortic bifurcation, has been established for over two decades [8], demonstrating acceptable patency rates and a good safety profile [9]. Building upon this technique, the Covered Endovascular Reconstruction of the Aortic Bifurcation (CERAB) approach was introduced to enhance outcomes in extensive AIOD, particularly for TASC-II C/D lesions [10]. CERAB entails proximal aortic stenting followed by bifurcational reconstruction with covered kissing stents. While early outcomes have demonstrated feasibility and safety, robust long-term data in complex patient cohorts remain limited [10].

The objective of this retrospective study was to evaluate the long-term efficacy and safety of endovascular reconstruction for advanced AIOD, classified as TASC-II C/D, over a 5-year follow-up period.

## 2. Materials and Methods

### 2.1. Study Design

This investigator-initiated, retrospective, single-center study included patients presenting with severe claudication or chronic limb-threatening ischemia (CLTI) (Rutherford categories 3–6), who underwent aortoiliac bifurcation reconstruction using either the kissing stent technique or the CERAB technique between December 2010 and December 2019. Eligible participants were aged ≥18 years and had AIOD classified as TASC-II C or D. No other exclusion criteria were applied; notably, acute symptom onset was not considered exclusionary. All patients provided written informed consent for inclusion in a prospectively maintained PAD database. The study was approved by the Institutional Review Board (approval no. 040/20 EK) and conducted in accordance with the Declaration of Helsinki.

### 2.2. Data Collection

Baseline patient characteristics were collected after standardized collection upon hospital admission from electronic medical records. These data included medical history, clinical presentation (including Rutherford classification), and current medication use. Lesion characteristics and procedural details were obtained from the interventional procedure report and angiographic imaging review. Aortoiliac lesions were categorized according to the TASC-II classification [3], and arterial calcification was graded using the Peripheral Artery Calcification Scoring System (PACSS) [11].

Following written informed consent, angiography was performed under local anesthesia. Vascular access was obtained under ultrasound guidance, with access site selection based on individual anatomy and arterial calcification, at the operator’s discretion. In cases requiring planned thromboendarterectomy (TEA) of the common femoral artery, general anesthesia was administered, and a hybrid surgical approach was employed in collaboration with the vascular surgery department.

Following vascular access, 5.000 units of intravenous heparin were administered. Both brachial and femoral access were used, at the discretion of the operator. After diagnostic angiography, reconstruction of the aortoiliac bifurcation was performed. The choice between the kissing stent technique and the CERAB technique was left to the discretion of the operator. Both methods have been previously described in detail [12,13]. Covered stents utilized in the procedures included LifeStream (Becton, Dickinson and Company, Franklin Lakes, NJ, USA), BeGraft (Bentley InnoMed GmbH, Hechingen, Germany), Advanta (Getinge AB, Wayne, NY, USA), Fluency (Becton, Dickinson and Company, Franklin Lakes, NJ, USA), and Gore Viabahn (W.L. Gore and Associates, Flagstaff, AZ, USA). Bare-metal stents (BMS) were additionally employed when necessary to reinforce the reconstruction. In selected cases, the chimney technique was used to treat obstructed visceral arteries and/or to preserve renal artery perfusion in cases of high aortic occlusions. Technical success was confirmed by final angiography and procedure-related and access site complications were documented until discharge.

### 2.3. Medication

Following covered stent implantation, patients received either dual antiplatelet therapy or an antiplatelet agent in combination with full-dose oral anticoagulation for a period ranging from 4 weeks to 3 months. In cases where there was an indication for therapeutic anticoagulation (e.g., atrial fibrillation), individualized antithrombotic regimens were established. For individuals not previously on antiplatelet treatment, a pre-procedural loading dose was given, consisting of 500 mg of acetylsalicylic acid (ASS) and/or 300 mg of clopidogrel.

### 2.4. Endpoints

Technical success was defined as a final residual diameter stenosis of ≤30% without evidence of device malfunction.

The efficacy endpoint was primary patency at 5 years, defined as the absence of target lesion revascularization (TLR) or binary restenosis, as assessed by duplex ultrasound demonstrating a peak systolic velocity ratio greater than 2.4. TLR was defined as any reintervention for a stenosis of ≥50% occurring within ±5 mm of the target lesion. Additionally, efficacy endpoints included freedom from TLR and secondary patency, defined as maintenance of target vessel patency through additional interventions for re-stenosis and/or re-occlusion. The safety endpoint was overall survival and freedom from major target limb amputation. Procedure and access site–related complications were also recorded.

### 2.5. Follow-Up

Patients underwent initial follow-up evaluation after 3–6 months, followed by annual follow-up evaluations over a 5-year period. Follow-up assessments included duplex ultrasound imaging, clinical symptom evaluation using the Rutherford classification, and measurement of the ankle–brachial index (ABI), conducted either on-site or by the outpatient angiologist. In cases where in-person follow-up data were unavailable, telephone interviews were conducted at the end of the follow-up period. These interviews assessed clinical status based on the Rutherford classification, the occurrence of TLR or major amputation of the target limb, and vital status. Patients were classified as lost to follow-up if two unsuccessful contact attempts were made by telephone and efforts to reach their primary physician also failed.

### 2.6. Statistical Analysis

Categorical variables are presented as absolute values and percentages, while continuous variables are reported as means with standard deviations (SD). Kaplan–Meier (KM) time-to-event analysis over a 5-year period was employed to assess primary and secondary patency, as well as freedom from TLR and overall survival. Primary patency was further analyzed according to implantation technique (kissing stent vs. CERAB) and comparisons between KM curves were performed using the log-rank test. Patients without events by the end of the follow-up period were censored at 1825 days.

A Cox proportional hazards regression model was fitted to identify factors associated with primary patency loss, TLR, and all-cause mortality (Supplemental Materials). The following variables were included: age, sex (male/female), number of target lesions, revascularization technique (kissing stent vs. CERAB), PACSS grade 3/4 (yes/no), TASC-II D classification (yes/no), presence of Leriche syndrome (yes/no), hypertension (yes/no), diabetes mellitus (yes/no), obesity (yes/no), hyperlipidemia (yes/no), history of smoking (yes/no), coronary artery disease (yes/no), chronic kidney disease (yes/no), previous intervention (yes/no), clinical presentation (1: claudication, 2: ischemic rest pain, 3: tissue loss, 4: acute onset), and perioperative risk (1: low risk = ASA II, 2: moderate risk = ASA III, 3: high risk = ASA IV–V). A stepwise selection model was used to avoid overfitting. Proportional hazards assumption was tested (*p* = 0.376) and multicollinearity was assessed using variance inflation factors (VIF; VIF < 2). Statistical significance was defined as *p* < 0.05. All analyses were conducted using SPSS Statistics version 29.0 (IBM Corp., Armonk, NY, USA) and R studio (Posit Software, PBC, Boston, Massachusetts, USA; version 2024.12.1 + 563).

## 3. Results

### 3.1. Patient and Lesion Characteristics

Between December 2010 and December 2019, 201 patients with AIOD underwent endovascular reconstruction of the aortic bifurcation. Patient characteristics are detailed in Table 1.

Cardiovascular risk factors were highly prevalent, with hypertension observed in 81.1% (163/201) and hyperlipidemia in 79.6% (160/201) of patients. The majority (79.6%, 160/201) were classified as American Society of Anesthesiologists (ASA) physical status stage ≥ 3, and 25.9% (52/201) of patients had a history of prior stent placement in the aortoiliac segment.

CLTI, characterized by rest pain or tissue loss, was present in 23.4% (47/201). Lesion and procedural data are summarized in Table 2.

All lesions were classified as TASC-II C or D, with 71.1% (143/201) categorized as TASC-II D lesions.

**Figure 1:**
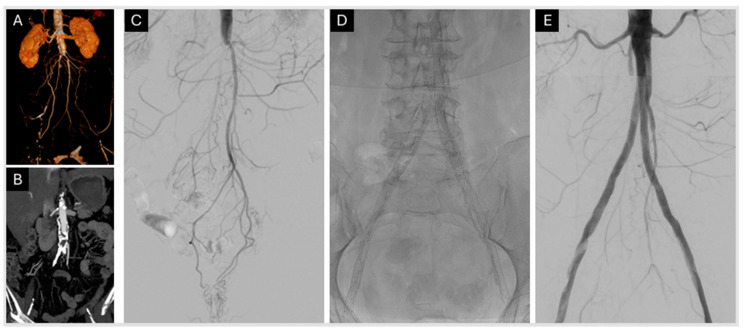
Case example using the kissing stent technique. (**A**) 3D reconstruction based on pre-interventional CT imaging; (**B**) pre-interventional CT demonstrating aortoiliac occlusive disease; (**C**) diagnostic angiography before intervention; (**D**) fluoroscopic image after reconstruction using the kissing technique; (**E**) final angiography showing the post-interventional result. CT, computed tomography.

**Figure 2:**
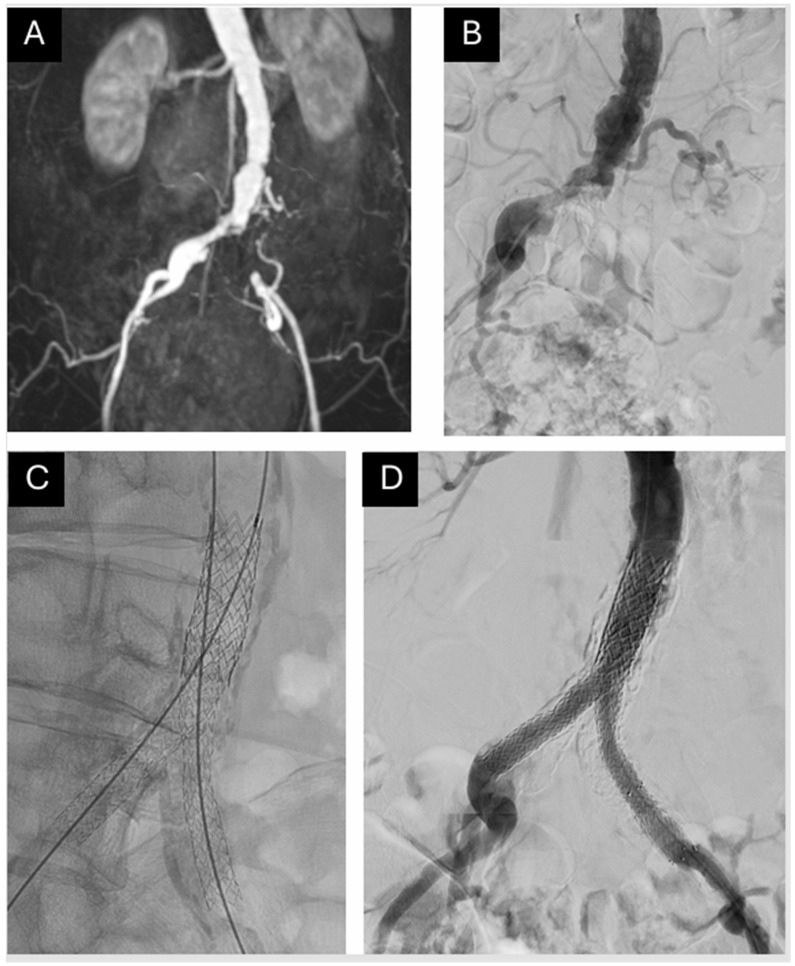
Case example using the CERAB technique. (**A**) Pre-interventional MR angiography; (**B**) diagnostic angiography before intervention; (**C**) fluoroscopic image after reconstruction using the CERAB technique; (**D**) final angiography showing the post-interventional result. CERAB: Covered Endovascular Reconstruction of the Aortic Bifurcation; MR, magnetic resonance.

The kissing stent technique (Figure 1) was employed in 87.1% (175/201) of procedures, while 12.9% (29/227) were treated using the CERAB technique (Figure 2).

Between 2010 and 2014, 83.0% (39/47) patients underwent aortic bifurcation reconstruction with kissing technique, while 17.0% (8/47) were treated with the CERAB technique. Between 2015 and 2019, 88.3% (136/154) patients underwent aortic bifurcation reconstruction with the kissing technique, while 11.7% (18/154) were treated with the CERAB technique. Technical success was achieved in 97.5% (196/201). The average hospital stay after the index procedure was 5.8 ± 4.9 days (range, 1–63).

Overall, major complications were reported in 6.5% (13/201) and access site-related complications in 3.0% (6/201) of cases. Details are presented in Table 3.

The in-hospital mortality rate was 1.5% (3/201) (Supplementary Table S1), with one death being classified as procedure-related. This patient initially presented with CLTI, with an occluded superior mesenteric artery and a stenotic celiac trunk. Following the intervention, the patient developed an acute abdomen. Postinterventional computed tomography angiography revealed complete occlusion of the celiac trunk with extensive mesenteric ischemia following infrarenal aortic reconstruction. Despite successful recanalization of the celiac trunk, the patient subsequently developed colon ischemia and multiorgan failure, resulting in death. This corresponds to a procedure-related mortality rate of 0.5% (1/201).

### 3.2. 5-Year Follow-Up

The mean follow-up duration was 1440.7 ± 591.8 days (range, 0–1825 days). Survival status at the 5-year follow-up was available for 79.6% of patients (160/201).

**Figure 3:**
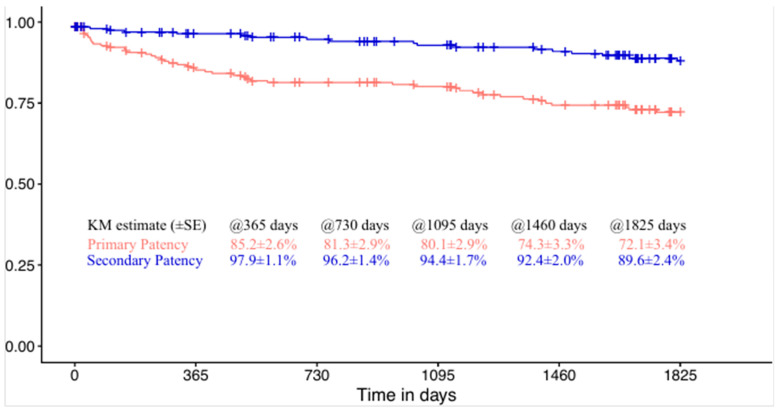
Kaplan–Meier estimates for primary (red) and secondary patency (blue). Kaplan–Meier estimates of primary patency and secondary patency. KM: Kaplan–Meier.

At the 5-year follow-up, KM estimates for primary patency were 72.1% ± 3.4% and 89.6% ± 2.4% for secondary patency (Figure 3).

**Figure 4:**
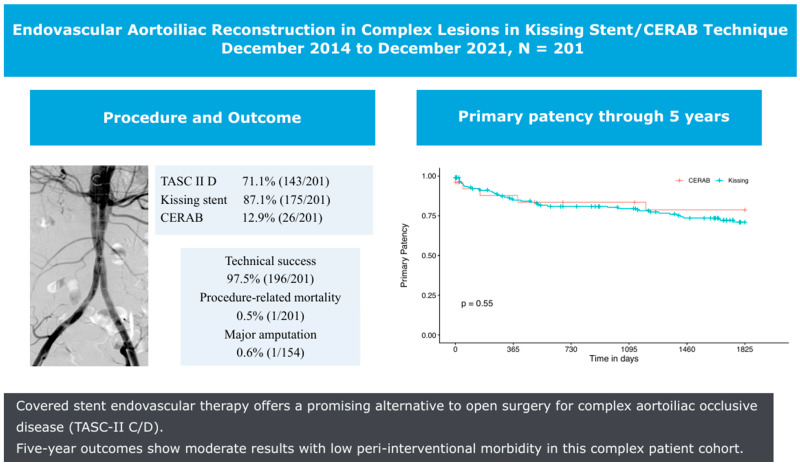
Final angiography after aortoiliac reconstruction with kissing stent technique (**left**), procedure characteristics, outcomes, and Kaplan–Meier estimates of primary patency over 5 years (**right**). CERAB: Covered Endovascular Reconstruction of the Aortic Bifurcation; TASC: TransAtlantic Inter-Society Consensus.

There was no difference in KM estimates of primary patency stratified between the kissing stent and CERAB technique (log-rank *p* = 0.55) (Figure 4).

To achieve secondary patency, 8 patients underwent TLR for re-stenosis (1725.9 ± 1083.6 days, range 83–2994 days) and 30 for re-occlusion (2031.4 ± 713.4 days, range 335–4003 days). KM estimates of freedom from TLR were 72.9% ± 3.4% at the end of the follow-up period (Supplementary Figure S1). The average improvement in the Rutherford category was 2.3 ± 1.3 after an observation period of 2 years. KM estimates of freedom from overall mortality were 79.9% ± 2.9% at 5 years (Supplementary Figure S2). Over the five-year period, only one patient (0.6%, 1/154) underwent major limb amputation.

### 3.3. Regression Analysis

In stepwise cox regression, the following observations were made. Regarding primary patency, older age (Hazard Ratio [HR] 0.96 per year; *p* = 0.01) was protective. Risk for loss of primary patency was significantly increased with diabetes (HR 2.61; *p* = 0.002), Leriche syndrome (HR 3.13; *p* < 0.001) and previous interventions in the aortoiliac segment (HR 3.14; *p* < 0.001). Regarding repeat TLR, similar results were observed, with older age (HR 0.96 per year; *p* = 0.019) being protective. Risk of TLR was significantly increased with diabetes (HR 2.77; *p* = 0.001), Leriche syndrome (HR 3.35; *p* < 0.001) and previous interventions in the aortoiliac segment (HR 3.44; *p* < 0.001).

Regarding overall mortality, risk was significantly increased with older age (HR 1.05; *p* = 0.002) and lung disease (HR 2.67; *p* = 0.008). High-risk patients (ASA 3 vs. ASA 1) demonstrated a significantly increased risk of all-cause mortality compared to those at low perioperative risk (hazard ratio [HR] 7.09; *p* = 0.002). In contrast, there was no statistically significant difference in all-cause mortality between moderate-risk and low-risk patients (HR 2.01; *p* = 0.26).

## 4. Discussion

Severe AIOD remains challenging, with lower technical success rates and a higher incidence of complication in heavily calcified lesions [14]. EVT has proven to be a safe and feasible option for the treatment of AIOD [10]. However, the optimal therapeutic approach remains under debate [15]. This study aimed to evaluate the long-term efficacy and safety of endovascular aortic bifurcation reconstruction in patients with TASC-II C/D lesions.

In our cohort, the majority presented with severe systemic comorbidities, placing them at elevated surgical risk and reinforcing the importance of an endovascular strategy in this population. Overall, the observed technical success was high and consistent with recent meta-analyses of the kissing stent and CERAB techniques, which report technical success rates exceeding 95% in complex aortic bifurcation reconstruction [9,10].

In this cohort, the primary patency rate was 72%, and the secondary patency rate approached 90%, with inclusion of patients presenting with acute limb ischemia to represent real-world clinical scenarios. In the ILIACS registry, patency outcomes were analyzed in 220 patients undergoing EVT of TASC II C/D AIOD with kissing stent reconstruction using either covered or bare-metal stents (n = 110 each). In that cohort, three-year primary and secondary patency rates were 92% and 98%, respectively, exceeding those observed in our cohort [16]. Consistent with our findings, older age was significantly associated with prolonged patency. Importantly, patients in our cohort were overall younger. A retrospective single-center study reported no significant differences in patency outcomes among patients with TASC II C/D lesions treated with kissing stents (n = 35), CERAB (n = 26), or a unibody bifurcated AFX stent graft (n = 26), with three-year primary and secondary patency rates of 84% and 99%, respectively, comparable to those in our cohort [17].

For the CERAB technique, a retrospective study of 160 patients, most treated for TASC-II C/D lesions, reported 5-year primary and secondary patency rates of 78% and 95%, respectively, comparable to those observed in our cohort [18]. Notably, only 25% of that cohort presented with CLTI, and patients with acute onset were excluded. In contrast, our cohort included a higher proportion of patients with CLTI and greater perioperative risk according to the ASA classification, reflecting a more advanced overall disease profile. In a study including 64 patients treated with kissing stenting or CERAB, 5-year primary and secondary patency rates were 70% and 77%, respectively, with CLTI present in only 20% [2]. A small study (n = 18) utilizing both techniques (77% kissing, 23% CERAB) in TASC-II C/D lesions showed favorable primary patency of 86%, though its small size may limit its generalizability [19].

In-hospital mortality and freedom from major target limb amputation were overall consistent with other reports [2,10,18]; however, there is limited data on long-term mortality. In a study comparing aortobifemoral bypass and kissing stenting in complex AIOD patients, long-term mortality was 16.5% at 6 years in the EVT group [20]. However, our patient cohort suffered from extensive comorbidities, which may explain the higher mortality over a follow-up period of 5 years. Of note, mortality in the open surgery group still surpassed our mortality rate with 35% [18]. In our study, one patient died from mesenteric ischemia resulting from celiac trunk occlusion after infrarenal aortic reconstruction. This has been reported in a previous study, highlighting the importance of evaluating the patency of the visceral arteries before aortoiliac reconstruction [2]. Periinterventional complications have been reported at around 10% in previous studies [2,9,10,21]. These findings are consistent with our results, with access-site related complications observed in 3% of cases.

In our cohort, patients with extensive AIOD requiring aortoiliac reconstruction were relatively young, with a mean age of 62 years. This trend is consistent with previous studies, which also reported predominantly younger patient cohorts [4,13,16,22]. In line with these findings, regression analysis identified older age as a protective factor against patency loss within our study population. In previous studies, younger age has also been identified as a significant risk factor in regression analyses of patients undergoing aortoiliac reconstruction for patency loss [9,23]. These findings reinforce existing evidence that suggests an aggressive form of vascular disease may be present in this young population [24]. Furthermore, regression analysis identified diabetes and Leriche syndrome as independent predictors of primary patency loss—all potential markers of multimorbidity and advanced disease progression. In addition, prior stent placement in the aortoiliac segment was found to be a significant risk factor, aligning with findings from previous regression studies [9,18]. As expected, older patients with severe comorbidities and advanced disease were at higher risk of mortality. Our study represents a large cohort focusing on the long-term outcomes of aortic reconstruction in patients suffering from AIOD. However, the retrospective design may introduce selection and information bias.

## 5. Conclusions

EVT has become a safe and efficient treatment option for AIOD classified as TASC-C or D lesions in patients with high-risk for general anesthesia, utilizing either the kissing stent or the CERAB technique. At 5 years, EVT demonstrated favorable patency rates in this complex cohort, with no significant difference between techniques and low perioperative morbidity and mortality. Younger patients demonstrated a higher risk of patency loss, reinforcing existing evidence that AIOD tends to be particularly aggressive in this population.

## Figures and Tables

**Table 1:** Patient characteristics.

Variables			N = 201
Demographics			
	Age		61.9 ± 9.7
	Female		61 (30.3)
	Body mass index (BMI > 30)		15 (7.5)
Clinical presentation			
	Rutherford category		
		3	154 (76.6)
		4	20 (10.0)
		5	25 (12.4)
		6	2 (1.0)
Medical history			
	Hypertension		163 (81.1)
	Dyslipidemia		160 (79.6)
	Diabetes type II		62 (30.8)
	Coronary artery disease		68 (33.8)
	Obstructive lung disease		23 (11.4)
	Smoking		164 (81.6)
		Never	37 (18.4)
		Current	119 (59.2)
		Former	45 (22.4)
	Renal insufficiency †		50 (24.9)
	Cerebrovascular disease		30 (14.9)
	ASA class		3.2 ± 0.8
		II	41 (20.4)
		III	119 (59.2)
		IV	40 (19.9)
		V	1 (0.5)
Previous treatment			
	Aortoiliac stent implantation		52 (25.9)
		Aorta	1 (0.5)
		CIA, unilateral	18 (9.0)
		CIA, bilateral	33 (16.4)
	Amputation		9 (4.5)

Data is given as n (%) or mean ± SD. † Renal insufficiency defined as eGFR < 60 mL/min/1.73 m^2^. ASA: American Society of Anesthesiologists. CIA: common iliac artery.

**Table 2:** Lesion and procedural characteristics.

Lesions				
	TASC-II classification *			
		C		58 (28.9)
		D		143 (71.1)
	Severe calcification †			51 (25.4)
	Target lesions overall			249
	Target lesions per patient			1.8 ± 0.6
		Aorta, stenosis		34 (13.7)
		Iliac arteries		215 (86.3)
			Stenosis	156 (72.6)
			Occlusion	59 (27.4)
Procedure				
	Access ‡			2.3 ± 0.5
		Ante- and retrograde		92 (45.8)
		Retrograde		109 (54.2)
	Stenting			
		Kissing technique		175 (87.1)
		CERAB technique		26 (12.9)
	Distal stent extension			111 (55.2)
		Unilateral		58 (51.2)
		Bilateral		53 (48.8)
	Number of stents			3.4 ± 1.8
		Ballon-expandable		364 (85.0)
		Self-expandable		64 (15.0)
	Stent length (mm)			
		Aorta		49.9 ± 14.2
		Iliac arteries		59.7 ± 29.5
	Stent diameter (mm)			
		Aorta		19.7 ± 4.6
		Iliac arteries		8.2 ± 1.0
	Visceral reconstruction			14 (7.0)
		Renal arteries		12 (6.0)
		SMA		3 (1.5)
	Inner stent strengthening with BMS			28 (13.9)
	Hybrid intervention with simultaneous TEA			14 (7.0)
	Technical success			196 (97.5)
Medication at discharge				
	DAPT			180 (90.0)
	Mono platelet inhibition + OAC			10 (5.0)
	DAPT + OAC (triple therapy)			10 (5.0)

Data is given as n (%) or mean ± SD. * TASC: TransAtlantic Inter-Society Consensus. † As defined by the Peripheral Artery Calcification Scoring System (PACSS); PACSS 3/4. ‡ Excluding the patients who were partially treated surgically with a hybrid procedure; n = 108, antegrade = brachial. CERAB: Covered Endovascular Reconstruction of the Aortic Bifurcation. SMA: Superior mesenteric artery. BMS: Bare-metal stent. TEA: Thromboendarterectomy. DAPT: Dual antiplatelet therapy. OAC: Oral anticoagulation.

**Table 3:** Major and access site complications.

Major complication				13 (6.5)
	Major bleeding			6 (3.0)
		Vessel perforation with covered stent implantation		2 (1.0)
		Transfusion		4 (2.0)
	Distal embolization (endovascular thrombectomy + lysis)			1 (0.5)
	Reperfusion syndrome			5 (2.5)
	Conservative treatment			3 (1.5)
		Fasciotomy		2 (1.0)
	Acute kidney injury			1 (0.5)
		AKIN II		0 (0)
		AKIN III		1 (0.5)
			New-onset dialysis	1 (1.3)
Access site complication				6 (3.0)
	Pseudoaneurysm, surgical revision			1 (0.5)
	AV-fistula, surgical revision			1 (0.5)
	Access artery stenosis/occlusion			4 (2.0)
		Surgical revision		3 (1.5)
		Endovascular revision		1 (0.5)

Data is given as n (%) or mean ± SD. AKIN: Acute kidney injury network. AV: Arteriovenous.

## Data Availability

The data presented in this study are available on request from the corresponding author. The data are not publicly available due to data privacy.

## Supplementary Materials

The following supporting information can be downloaded at https://www.mdpi.com/article/10.3390/jcm15145409/s1: Table S1: In-house mortality. Figure S1: Survival curve and numbers at risk for freedom from target lesion revascularization. TLR: Target lesion revascularization. Figure S2: Kaplan–Meier survival curve and numbers at risk for freedom from all-cause mortality.

## Abbreviations

The following abbreviations are used in this manuscript:

EVT	Endovascular therapy
AIOD	Aortoiliac occlusive disease
CERAB	Covered Endovascular Reconstruction of the Aortic Bifurcation
KM	Kaplan–Meier
PAD	Peripheral artery disease
TASC	TransAtlantic Inter-Society Consensus
CLTI	Chronic limb-threatening ischemia
PACSS	Peripheral Artery Calcification Scoring System
BMS	Bare-metal stents
TLR	Target lesion revascularization
ABI	Ankle–brachial index
ASA	American Society of Anesthesiologists
