# Supplementary material for: Five-Year Outcomes of Endovascular Aortoiliac Reconstruction in Complex Lesions: A Retrospective Single-Center Study
Source: J Clin Med. 2026 Jul 10;15(14):5409. doi: 10.3390/jcm15145409 (PMC13410659; doi:10.3390/jcm15145409)

# Supplementary Material

## Supplemental Table S1. In-house mortality

| | |
|---|---|
| In-hospital Mortality | 3 (1.5) |
| Sepsis due to pre-existing ulceration (Rutherford 6) | 1 (0.5) |
| Multiorgan failure after endovascular procedure | 2 (1.0) |

**Supplemental Figure S1.** Survival curve and numbers at risk for freedom from target lesion revascularization. TLR: Target lesion revascularization.

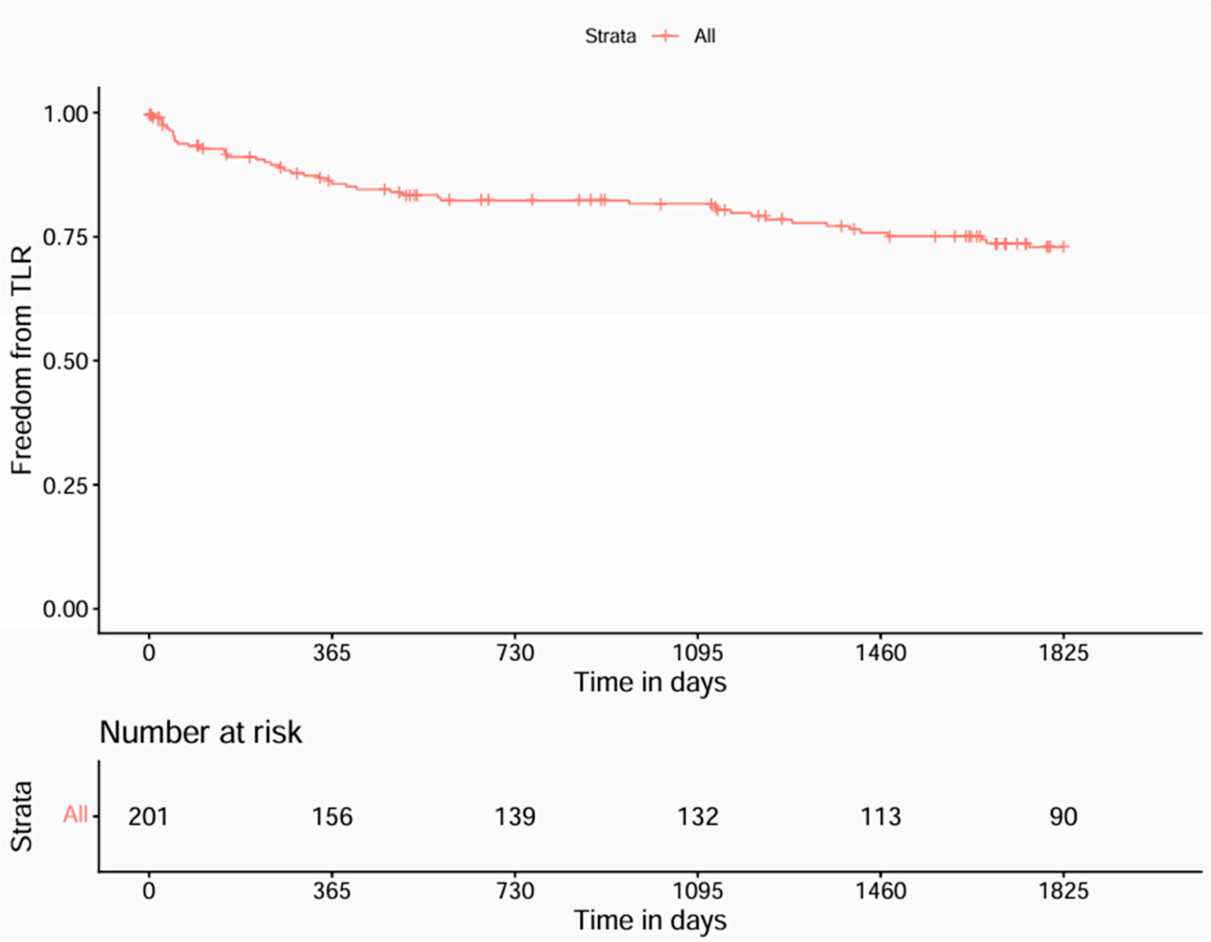

**Supplemental Figure S2.** Kaplan Meier survival curve and numbers at risk for freedom from all-cause mortality.

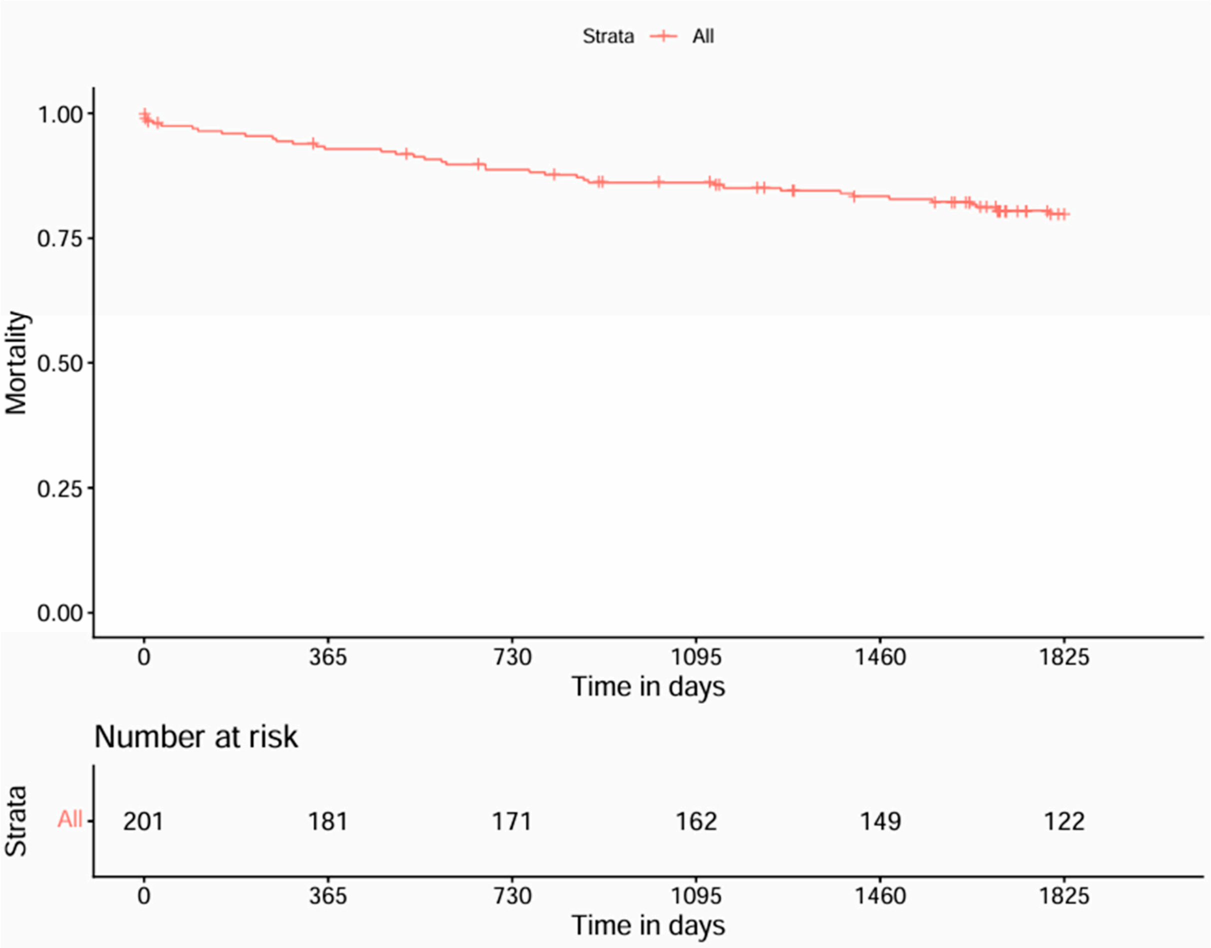

Supplement: Supplementary file 1 [file jcm-15-05409-s001.zip › jcm-4297163-supplementary.pdf]
